# A system theory based digital model for predicting the cumulative fluid balance course in intensive care patients

**DOI:** 10.3389/fphys.2023.1101966

**Published:** 2023-04-13

**Authors:** Mathias Polz, Katharina Bergmoser, Martin Horn, Michael Schörghuber, Jasmina Lozanović, Theresa Rienmüller, Christian Baumgartner

**Affiliations:** ^1^ Institute of Health Care Engineering with European Testing Center of Medical Devices, Graz University of Technology, Graz, STM, Austria; ^2^ CBmed Center for Biomarker Research in Medicine, Graz, STM, Austria; ^3^ Institute of Automation and Control, Graz University of Technology, Graz, STM, Austria; ^4^ Department of Anesthesiology and Intensive Care Medicine, Medical University of Graz, Graz, STM, Austria

**Keywords:** fluid balance, system theory, transfer function model, prediction, intensive care, decision support

## Abstract

**Background:** Surgical interventions can cause severe fluid imbalances in patients undergoing cardiac surgery, affecting length of hospital stay and survival. Therefore, appropriate management of daily fluid goals is a key element of postoperative intensive care in these patients. Because fluid balance is influenced by a complex interplay of patient-, surgery- and intensive care unit (ICU)-specific factors, fluid prediction is difficult and often inaccurate.

**Methods:** A novel system theory based digital model for cumulative fluid balance (CFB) prediction is presented using recorded patient fluid data as the sole parameter source by applying the concept of a transfer function. Using a retrospective dataset of n = 618 cardiac intensive care patients, patient-individual models were created and evaluated. RMSE analyses and error calculations were performed for reasonable combinations of model estimation periods and clinically relevant prediction horizons for CFB.

**Results:** Our models have shown that a clinically relevant time horizon for CFB prediction with the combination of 48 h estimation time and 8–16 h prediction time achieves high accuracy. With an 8-h prediction time, nearly 50% of CFB predictions are within ±0.5 L, and 77% are still within the clinically acceptable range of ±1.0 L.

**Conclusion:** Our study has provided a promising proof of principle and may form the basis for further efforts in the development of computational models for fluid prediction that do not require large datasets for training and validation, as is the case with machine learning or AI-based models. The adaptive transfer function approach allows estimation of CFB course on a dynamically changing patient fluid balance system by simulating the response to the current fluid management regime, providing a useful digital tool for clinicians in daily intensive care.

## 1 Introduction

Fluid management is a challenging part of intensive care unit (ICU) treatment. In this regard, monitoring of vital signals and periodic assessment of fluid status are crucial. In daily clinical practice, the amount of body fluids gained or lost is usually estimated by calculating the external daily fluid balance (Shepherd, 2011). This relates the amount of fluid administered during the day to the fluid losses observed during the same period. For long-term monitoring of fluid status, calculation and visualization of vital signs in combination with the cumulative fluid balance (CFB) can be beneficial. The CFB is calculated as *cumulative fluid intake (CFI)* minus *cumulative losses (CFL)* over ICU stay.

**FIGURE 1:**
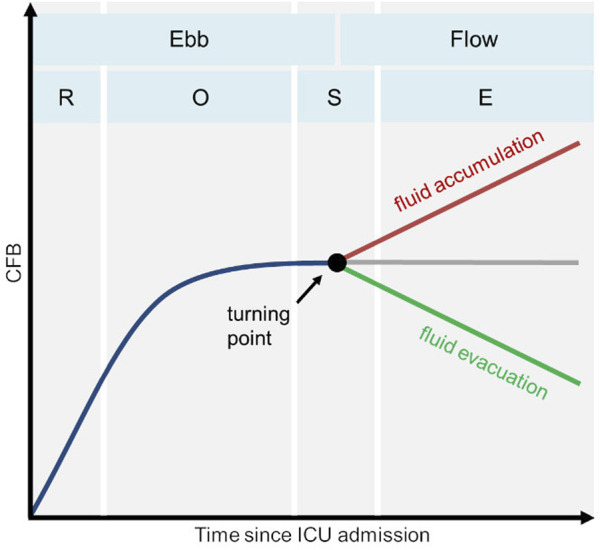
Possible trajectories of cumulative fluid balance (CFB) in postsurgical patients admitted to the intensive care unit (ICU) during the four consecutive phases of fluid therapy (Hoste et al., 2014; Malbrain et al., 2014; 2018; Ogbu et al., 2015). Rescue (R), optimization (O), stabilization (S), evacuation (E).

Possible CFB courses during a patient’s stay in the ICU have been described previously for critically ill, hyperhydrated patients (Hoste et al., 2014; Malbrain et al., 2014; 2018; Ogbu et al., 2015) and can be divided into four successive phases, as shown in Figure 1. In the first two phases, fluid accumulation occurs primarily, as demonstrated by a steadily increasing CFB due to administration of high volumes of resuscitation fluids and low fluid losses. Once hemodynamic stabilization is achieved, the patient’s fluid status may recover spontaneously in the subsequent phases and the excess volume can be evacuated. Otherwise, active fluid management strategies may be considered (Goldstein et al., 2014; Rosner et al., 2014; Claure-Del Granado and Mehta, 2016; Monnet et al., 2016) to restore euvolemia, as, for example, a high fluid overload is associated with severe side effects (Malbrain et al., 2018; Ouchi et al., 2020) and an increased risk of death (Rosenberg et al., 2009; Lee et al., 2015; Messmer et al., 2020). Especially after cardiac surgery, liberal fluid therapy has been shown to lead to higher in-hospital mortality and cardiovascular complications (Palomba et al., 2022).

Based on the calculations for the daily fluid balance, individual goals are usually set for the patient to achieve optimal fluid management during the continued stay in the ICU. However, these goals may change due to alterations in the patient’s health status and the resulting adjustments to ongoing therapy. Therefore, setting adequate and achievable fluid balance goals requires profound and experienced knowledge. In addition to an intake-output-calculation, both currently applied therapies and planned interventions must be considered to estimate a specific fluid balance goal. Therefore, assessing and predicting the fluid balance can be challenging.

Recent approaches to predict various physiological parameters using artificial intelligence (Johnson et al., 2018; Gutierrez, 2020), deep or machine learning have been developed and applied in various clinical settings (Celi et al., 2008; Komorowski et al., 2018; Li et al., 2018; Parreco et al., 2018; Rojas et al., 2018; Giannini et al., 2019; Shillan et al., 2019). However, model building using these methods requires previously acquired training data collected in large study populations. In this work, we develop and evaluate a system and control theory based model to support fluid management in postsurgical patients without requiring a prior data set for model training. In addition to modeling the hemodynamic response (Chen et al., 2004; Bighamian et al., 2018), the use of control techniques, particularly closed-loop approaches, have been described previously, e.g., in intraoperative situations (Dumont, 2012; Miller and Gan, 2013; Rinehart et al., 2014; Joosten et al., 2015; 2016; 2019; Restoux et al., 2016) and ventilation (Sanchez-Morillo et al., 2017; Kwong et al., 2019; Radhakrishnan et al., 2019), maintaining organ perfusion in brain death (Soltesz et al., 2018), fluid resuscitation (Kramer et al., 2008; Bighamian et al., 2016; Hundeshagen et al., 2017), sepsis (Merouani et al., 2008; Uemura et al., 2017), ICU sedation control (Haddad and Bailey, 2009; Gholami et al., 2012; Padula et al., 2017), in the management of diabetes (Hovorka, 2011; Haidar, 2016; Thabit et al., 2017; Kovatchev, 2018), in antibiotics administration (Herrero et al., 2018), in detecting the onset of seizure (Kamali et al., 2020) and in gait analysis (Nacpil et al., 2021).

In this work, we present a first-of-its-kind digital model for predicting the patient-specific CFB trend over clinically relevant therapeutic timespans based on single patient CFI and CFB time series data. The algorithm should not only serve to improve the adjustment or optimization of therapies after cardiac surgery, but also to reduce the workload in intensive care units, as estimating the current CFB course is very time-consuming. The control theory model is intended to provide a basis for a more generally applicable algorithm.

## 2 Materials and methods

### 2.1 Study population and sample selection

A retrospective data set with a total of *n* = 2061 adult patients collected between 2011 and 2017 was used for sample selection. All patients underwent elective cardiac surgery for coronary artery bypass grafting and/or surgical valve replacement with subsequent admission to the cardio-thoracic intensive care unit at the Medical University of Graz, Austria. The study was approved by the Ethics Committee of Medical University of Graz in accordance with the Declaration of Helsinki (vote EK 30-076 ex17/18).

**FIGURE 2:**
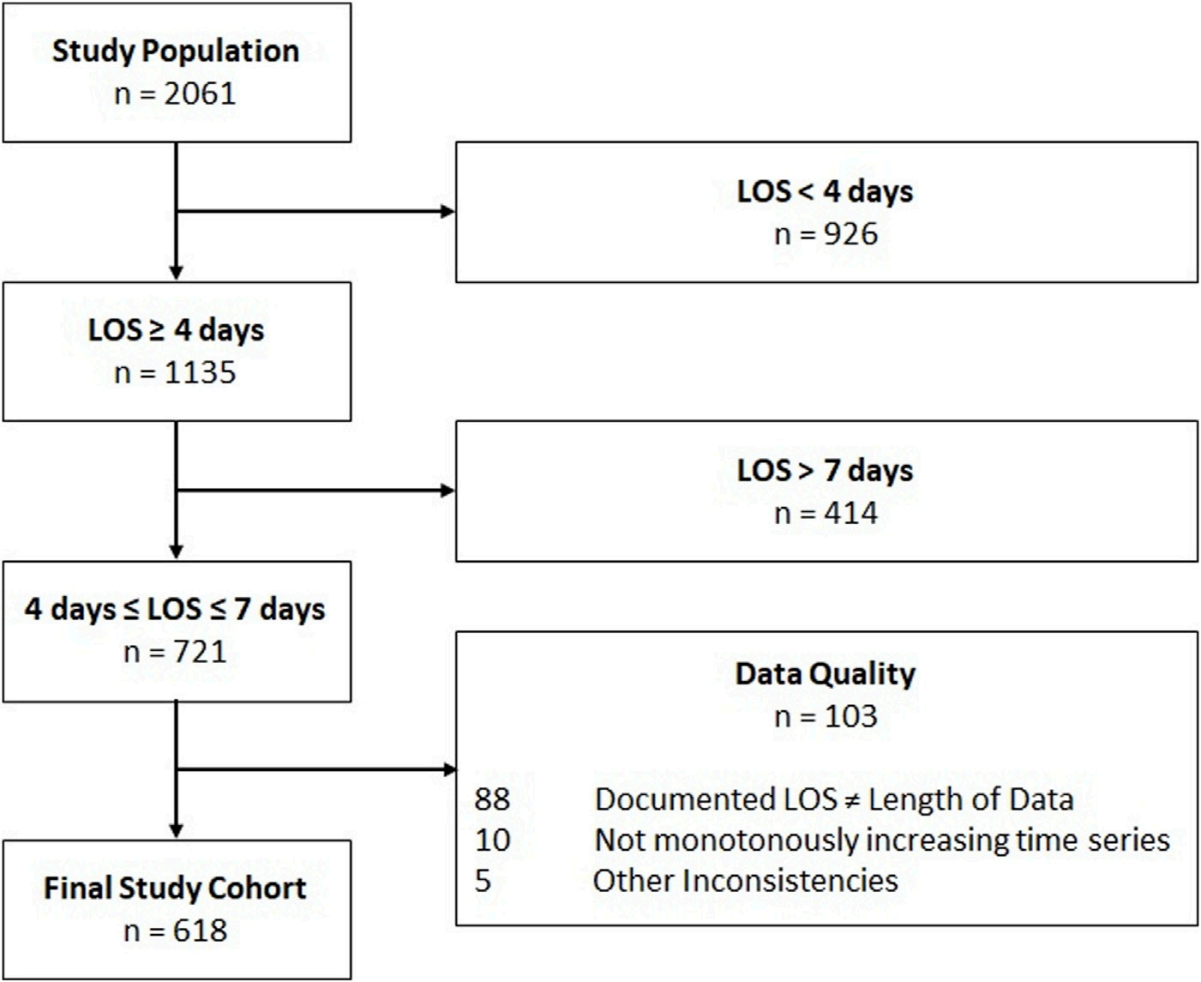
Overview of exclusion criteria for obtaining the final study cohort. Length of stay (LOS).

*n* = 926 patients were excluded because length of stay was less than 4 days and hemodynamic stabilization after surgery takes approximately 3 days (Hill and Hill, 1998; Desborough, 2000). *n* = 414 p atients with ICU length of stay greater than 7 days were also excluded, as they tended to have higher mortality and risk for readmission (Carden et al., 2008). This criterion had to be taken into account to increase the homogeneity to validate the algorithm in patients whose fluid trajectory corresponds to the previously described four phase model of fluid therapy. The re-accumulation of fluids after an evacuation phase caused by instabilities and the need for re-evacuation was not considered in this study. After a final quality check of the data (availability of fluid data for the entire stay, inconsistent data entries such as negative fluid intake), patient selection resulted in a subset of *n* = 618 patients as can be seen in Figure 2. Patient-related data and medication extracted from the electronic health record system are summarized in Table 1.

**TABLE 1:** Patient characteristics of the study population. Coronary artery bypass grafting (CABG), surgical valve replacement (SVR), length of stay (LOS), liters (L), cumulative fluid balance (CFB), Simplified Acute Physiology 3 Score (Moreno et al., 2005) (SAPS3), standard deviation (SD).

Study population	*n* = 618
Age (y)*	69 ± 10
Weight (kg)*	79 ± 19
Female	189 (30%)
LOS (d)*	5.00 ± 1
Type of operation	
CABG	40.6%
SVR with CABG	18,6%
SVR	40.8%
CFB at end of stay (L)*	1.67 ± 2.81
SAPS3	38.49 ± 7.97

* mean ± SD.

### 2.2 A system theory based digital model for predicting fluid balance

A fundamental concept within system theory is the application of a transfer function (TF), which is often used to describe the behavior of a physical or biological system. The TF allows the analysis of the dynamic properties of a system that models the systems output for each possible input function.

In the discrete-time domain, the TF can be determined using a discrete-time input time series U[k] and the corresponding output time series Y[k]. The Z-transforms of both signals (U[z] and Y[z]) are related to give the TF of the patient, P[z]. Following the control system theory, the transfer function should be chosen to correspond to the lowest-order differential equation to describe the behavior of a system. Basically, from a physiological point of view, oscillation and instability of the response to a fluid input is possible, resulting in the model to be at least a second order system. However, higher order systems led to a worse prediction in general. Possible reasons could be overfitting or the low variability of the input data, which makes an identification of a system depending on multiple parameters more difficult. Since it is not possible to increase the variability of the input data artificially, the transfer function with the empirically determined best result in prediction was selected. The model can basically be compared to a physical two tank water level system. The exponent n (see Eq. 1) that determines the order of the TF was set to *n* = 2. The general mathematical model of the system within the Z-domain is given by Eq. 1. The output of the patient model can never depend on a future input, so the condition m ≤ n must hold for a causal system.

$$P\left[z\right]=\frac{Y\left[z\right]}{U\left[z\right]}=\frac{\sum_{k=0}^{m}b_{k}z^{k}}{\sum_{k=0}^{n}a_{k}z^{k}}$$ (1)

**FIGURE 3:**
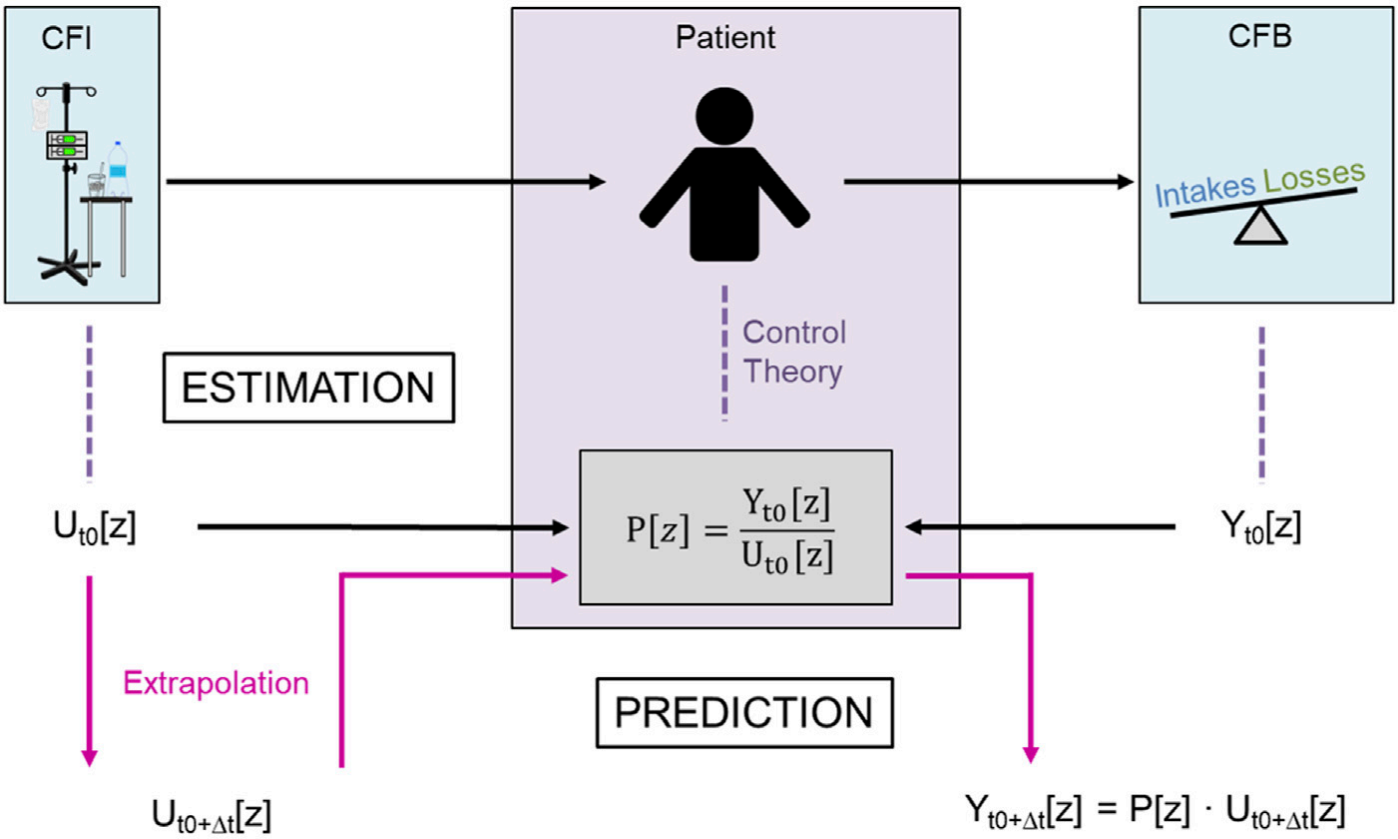
Overall approach to predicting the course of cumulative fluid balance (CFB) using the cumulative fluid intake (CFI) as the only input parameter.

The basic idea of CFB prediction using a system theory based modeling approach is shown in Figure 3. The process can be divided into two major steps: (i) estimation of a linear model of the patient’s response to fluid intake using a TF in the Z-domain based on the discrete-time CFI as the system input function and the discrete-time CFB as the output function of the system, and (ii) prediction of the future CFB trajectory based on the patient’s individual TF model and constant fluid intake. This so-called “black box” problem reduces the patient’s CFB response to fluid intake to a simple relationship between CFI and CFB expressed by the TF, which is used as a mathematical model to estimate the future CFB course under the clinical assumption that a patient’s treatment remains unchanged during the prediction period.

### 2.3 Application of the model

For model implementation and prediction MATLAB (version 9.9 - R2020b Update 1 on Windows 10) and the system identification library (version 9.13) were used. The “iddata” function defines the transposed CFI and CFB as a system object. The number of poles of the TF model P[z] was set to 2, the number of zeros to 1, the sampling time was set to 60 s. By entering the “iddata” object and selected parameters into the “tfest” function, a TF object is created by non-linear least-squares search based updates to minimize a weighted prediction error norm. As the CFB is directly calculated from the CFI and CFL there is no delay from input to output assumed and therefore feedthrough must be activated by setting the “Feedthrough” parameter of “tfest” true. This object, along with the modeled and extrapolated input function, was used to simulate and predict the CFB trajectories. The resulting estimated TF with the previously described defaults can be seen in following Eq. 2. Due to the activated feedthrough, the numerator is extended by the zero leading coefficient b_0_.

$$P\left[z\right]=\frac{{b_{0}+b}_{1}z^{-1}}{1+a_{1}z^{-1}+a_{2}z^{-2}}$$ (2)

#### 2.3.1 Preprocessing of raw fluid data

Fluid data was imported from an SQL data base and included intakes (colloid fluids, crystalloid fluids, medication fluids, oral intakes, parenteral nutrition, blood products) and losses (urine, drainage, renal replacement therapy, vomiting, blood samples, stool, miscellaneous) with their corresponding time points. The cumulative time series data were interpolated individually dependent on the specific type of administration or type of fluid loss using the “interp1” function for the entire length of stay at a sampling rate of one data point per minute. Interpolation was performed by inserting the last available value that followed the cumulative shape of the fluid data.

Gravity infusions that did not have a documented ending timestamp were assumed by clinicians with a flow rate of about 16 mL per minute until the total administered volume was reached. Short infusions and drinking were included as bolus. Insensible losses such as estimating the volume lost by sweating were not considered in the data. Urine data was linearly interpolated to the next available documented entry with the simplified assumption that the bladder has filled evenly in a linear fashion during this time.

**FIGURE 4:**
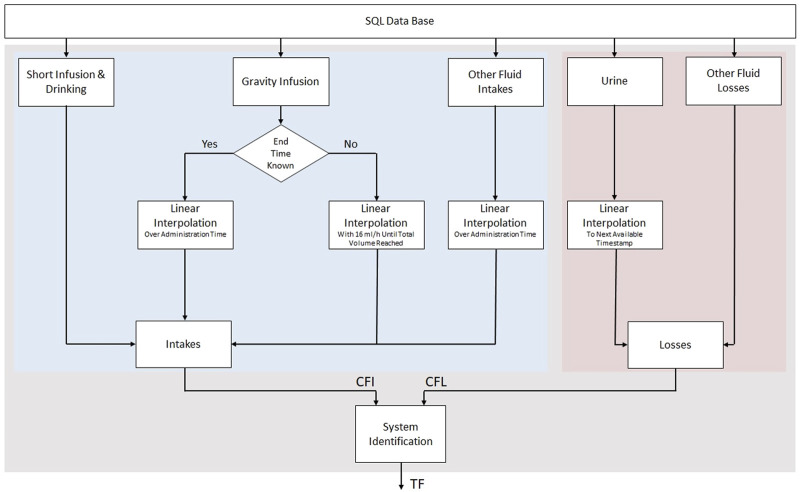
Flow graph of single patient data fusion and preprocessing for estimating a patient specific TF using the interpolated CFI and CFL data.

The resulting individual CFI and CFL time series were subtracted to calculate the corresponding CFB to identify the TF. The described procedure for preprocessing the input and output data can be seen in detail in the flow chart in Figure 4.

#### 2.3.2 Model estimation using the sliding window approach

Since fluid balance is constantly changing due to dynamically changing patient characteristics, the TF has to be periodically recalculated. A sliding window approach was chosen for TF estimation for (i) computational reasons and (ii) clinical considerations, because patients may become unstable again, which affects subsequent estimates. The length of the sliding window was set at 48, 72, 96, 120 and 144 h, with the first 24, 48, 72, 96 and 120 h used for TF estimation (*estimation time*) and the last 24 h of each window used for prediction (*prediction time*). The sliding window was incrementally shifted by 2 h. Table 2 shows the number of patients with sufficient length of stay to provide data for a given estimation time plus prediction time. Because the sliding window approach allows multiple predictions per patient, the total number of predictions is also shown.

**TABLE 2:** Number of predictions per estimation time.

Estimation time	Number of patients	Number of predictions
24	618	20,915
48	618	13,499
72	404	6,320
96	197	2,114
120	44	262

Data in the estimation time period preprocessed as uniformly sampled data is used to generate the discrete-time transfer function with the determined poles and zeros. The offset of the CFB is always set to 0 as initial condition for every window.

#### 2.3.3 CFI extrapolation

For prediction, the future CFI has to be estimated by extrapolation of the current fluid therapy or available data for the planned administered volumes. To provide a simple and fast method for CFI trend analysis and identification of paradigm shifts within a given time series without knowing future fluid therapy, the CFI for all estimation time windows was modeled using the piecewise.linear function of the SiZer package in R (Sonderegger, 2020). The function allows for a “broken-stick” model, where two lines are connected at a previously unknown point in time, thus providing a simple and fast method for identifying turning points within a given time series—in particular the point in time at which the CFI noticeably decreases (Toms and Lesperance, 2003). Extrapolation of the approximated CFI is performed using the predict function (base R) based on the piecewise linear model. We extrapolated the CFI model data for 24 h, which corresponds adequately to a clinically relevant time frame from ward visit to ward visit. This modeling and extrapolation step is required for applicable TF-based prediction.

#### 2.3.4 Statistical analysis of model predictions

Data preprocessing and statistical analyses were performed in *R* (version 4.0.2) within *RStudio* (version 1.3.959, RStudio PBC, Massachusetts, United States). The simulated (predicted) CFB course was compared with the real CFB within the maximal prediction period of 24 h. The root mean squared error (RMSE) of the last 30 min between both curves was calculated to determine the change of prediction error over time for the selected prediction times of 8, 12, 16, 20 and 24 h. This results in different prediction error combinations for model estimation (24–120 h) and model prediction (8–24 h) for a patient. Depending on the length of stay of each patient, not all combinations were available for all patients (see also Table 2). By using a sliding window, multiple predictions per patient could be obtained for most combinations.

## 3 Results

**FIGURE 5:**
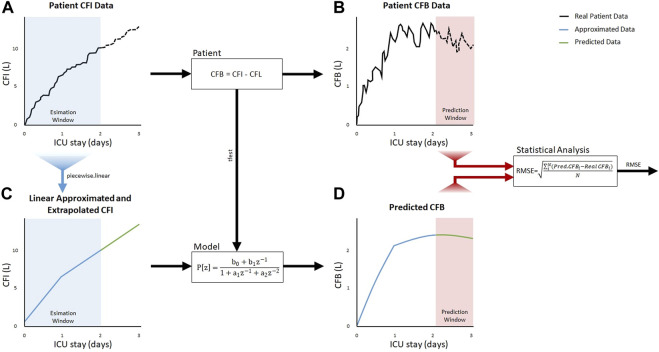
System theory based model applied to a selected single patient showing the estimation window 0–48 h after ICU admission in blue, which was used to determine the transfer function P[z] with the measured CFI and calculated CFB patient data in the upper row. The left column **(A, C)** shows the approximation and extrapolation of the cumulative fluid intake (CFI) used to predict the cumulative fluid balance (CFB) on the bottom right **(D)**. Measured future patient CFB **(B)** and predicted CFB **(D)** in the prediction window was used for statistical analysis of the RMSE.

The clinical applicability of the proposed model is first demonstrated exemplarily in a single patient (see Figure 5) and then evaluated in a homogeneous study population of cardiac intensive care patients.

### 3.1 Single patient

The selected example patient was originally admitted to the ICU for 2 days. The entire 2-day stay was chosen as estimation time for identifying the patient’s TF. After generating the TF P[z] using the measured CFI (Figure 5A) and calculated CFB data (Figure 5B) 0–48 h after ICU admission, the TF model was then used to predict the patient’s future CFB course. Here, the CFI time series data were linearly approximated and extrapolated by 24 h which can be seen in Figures 5A, C and was then applied to the patient’s previously calculated TF, resulting in a prediction of the CFB course for the next 24 h (Figure 5D). To verify the prediction, the RMSE between the predicted CFB and the original acquired time series data was calculated after 48 h (RMSE = 0.207).

### 3.2 Study cohort

**FIGURE 6:**
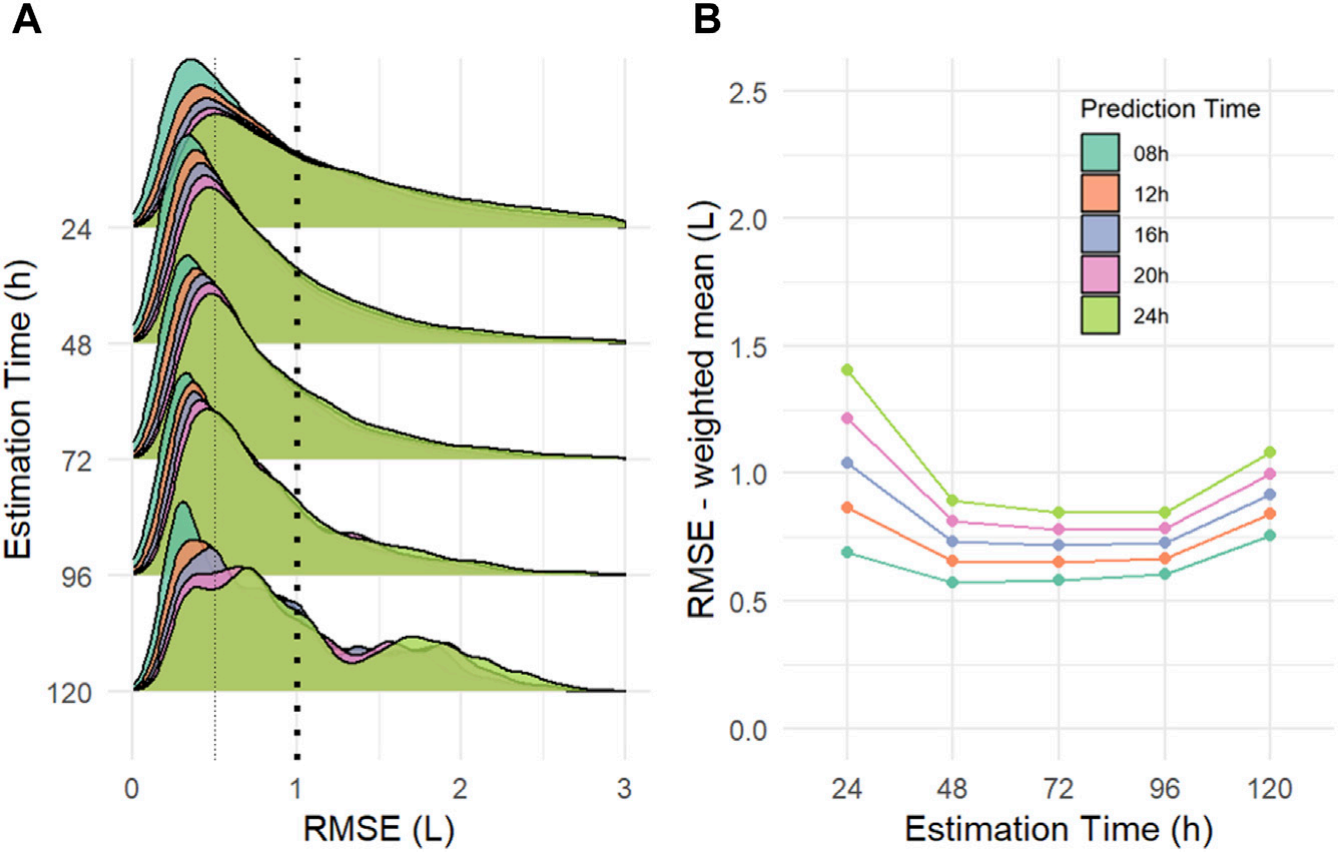
**(A)** Distributions of aggregated root mean squared error (RMSE) for the entire study cohort (*n* = 618) for different combinations of estimation and prediction time spans in liters (L) and hours (h), respectively. **(B)** Weighted mean values of aggregated RMSE for different combinations of estimation and prediction time spans in liters (L) and hours (h), respectively.

To evaluate the performance and predictive ability of our method, a total of *n* = 618 cardiac intensive care patients were analyzed retrospectively. Figure 6A shows the distributions of aggregated patient RMSE values for the entire cohort, plotted for the different combinations of estimation and prediction time spans. The overall prediction error decreases already after extending the estimation time from 24 to 48 h, whereas further extension shows only smaller changes. This result is best seen with longer prediction time periods. Figure 6B depicts the weighted mean values of the aggregated RMSE of the study population for different combinations of estimation and prediction periods.

**FIGURE 7:**
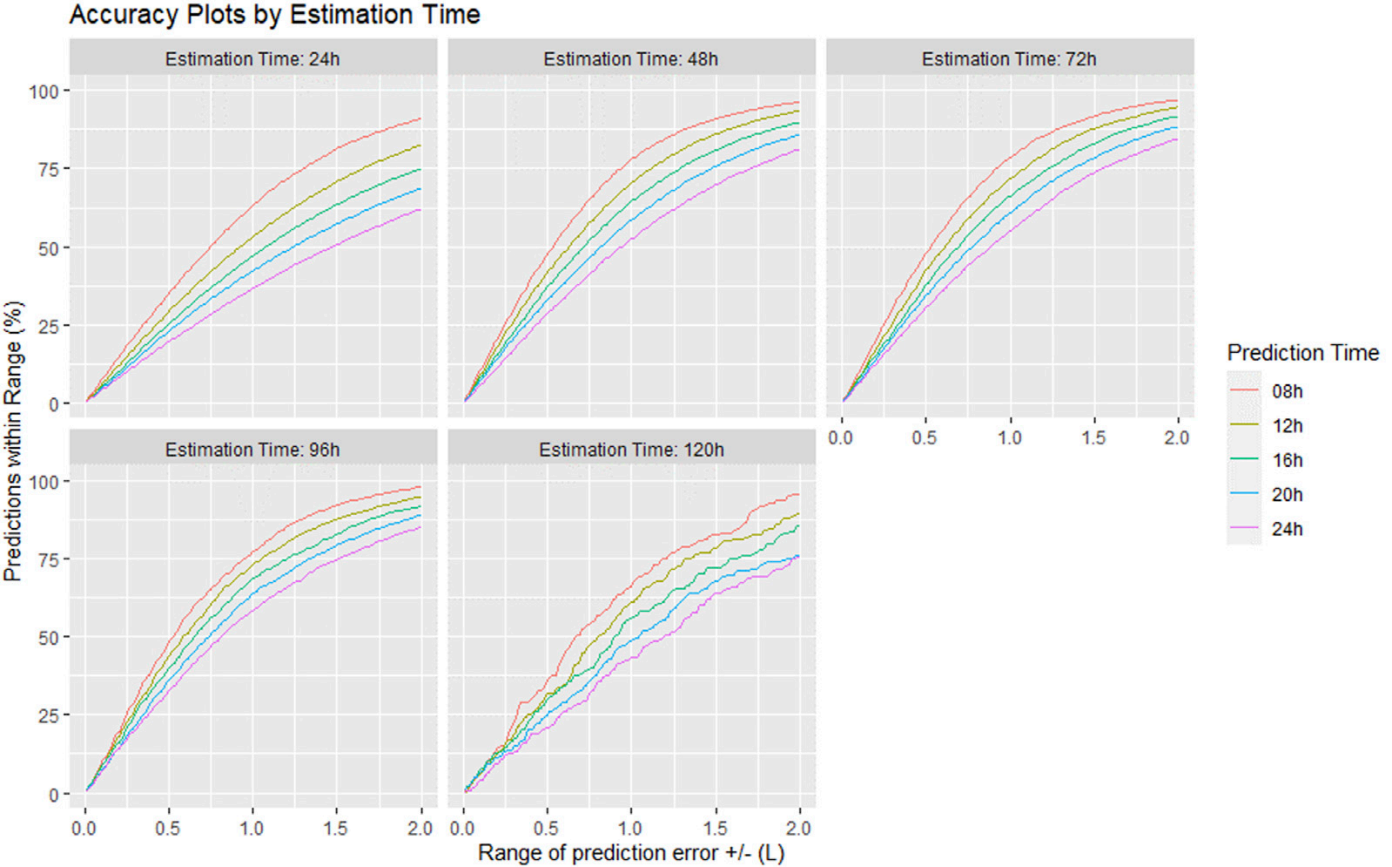
Number of predicted cumulative fluid balance values at the end of the prediction period in percent (%) within specified prediction error ranges in liters (L) for various combinations of estimation and prediction times.

Figure 7 shows the number of predictions included in each error ranges for different combinations of estimation and prediction times. The limit of maximal 2 L (L) for the error intervals was chosen by clinicians based on considerations of applicability in clinical practice, since there are currently no uniform (consensus) medical guidelines. The clinically relevant time horizon for predicting CFB with high accuracy results from the combination of 48 h estimation time and 8–16 h prediction time. This means that at a prediction time of 8 h, almost 50% of CBF predictions are within the range of ±0.5 L, and 77% of predictions are within the range of ±1.0 L. For longer prediction times (e.g., 16 h) these values decrease to 37% (±0.5 L) and 63% (±1.0 L), respectively.

## 4 Discussion

In cardiac intensive care, fluid administration must be used with caution and tailored to the current needs of the individual patients (Bignami et al., 2017). Conventional postoperative fluid therapies are applied primarily for replacement, maintenance, and nutrition purposes (Van Regenmortel et al., 2018) and often deliver large volumes of various fluids to the critically ill patient (Hessels et al., 2016). Despite skepticism (Gonzalez and Vincent, 2011; Perren et al., 2011; Cronhjort et al., 2016; Köster et al., 2017; Davies et al., 2019), fluid balance could be a valuable biomarker (Bagshaw et al., 2008) for assessing fluid status and estimating mortality risk (Epstein and Peerless, 2006; Bouchard et al., 2009; Rosenberg et al., 2009; Boyd et al., 2011; Grams et al., 2011; Vaara et al., 2012; Micek et al., 2013; Teixeira et al., 2013; Barmparas et al., 2014; Acheampong and Vincent, 2015; de Oliveira et al., 2015; Elofson et al., 2015; Sirvent et al., 2015; Brotfain et al., 2016; Genga and Russell, 2016; Marik et al., 2017; Pittard et al., 2017; Sakr et al., 2017; Chao et al., 2018; Codes et al., 2018; Li et al., 2018; Shen et al., 2018; van Mourik et al., 2019).

Cardiac surgery patients usually require large volumes of resuscitation fluids to compensate for the perioperative fluid loss. Due to the high amount of fluids, this group was selectively used as the first validation cohort of the algorithm but is not representative for other surgical interventions. We hypothesized that complications lead to an increased length of stay which we used as an exclusion criterion for the subgroup to narrow down the cohort to stable courses and therefore form the basis of a more complex system theoretical model that may be applied commonly for postoperative fluid management.

To depict a reliable CFB course, both fluid intake and fluid output must be accurately recorded. However, small volumes in particular may remain undocumented (Bashir et al., 2017), which—among other sources of error—may compromise the effectiveness of fluid balance monitoring. Several evaluations suggest that 35% of fluid data documented in patients’ medical records are inaccurate or missing (Reid et al., 2004; Perren et al., 2011; Diacon and Bell, 2014; Asfour, 2016; Davies et al., 2019; Lim et al., 2021), leading to a biased representation of patient characteristics and inaccurate assessment of the current CFB trend when only daily changes are considered.

To provide clinicians with a model that adequately represents the patient characteristics based on the CFB trajectory and predicts the future CFB course, we proposed an initial digital model based on system and control theory that can be divided into two parts. First, creating a mathematical representation of a patient’s fluid balance characteristics in the critical care situation and second, predicting the patient’s future CFB course to help clinicians optimize therapeutic fluid management. Here, the “system” status of each patient is mathematically modeled by a discrete-time second-order TF determined from the past CFI and CFB courses of limited lengths, i.e., the estimation time of the model, using a sliding window approach.

Only the CFI data up to the time of prediction were used for identifying the transfer function. Extrapolating the actual trend rather than predicting a specific value at the end of the prediction window by using the estimated future fluid intake as input for the TF emphasizes clinical applicability. The alternative measure of predictive accuracy using RMSE was again performed for all combinations of prediction and estimation time to provide a simple and intuitive interpretation for the user and a more practical approach for daily clinical practice. The prediction accuracy of this measure is reported as percentage of predictions within a maximum deviation of 2 L, which was considered by the clinicians in this study to be the upper limit of tolerable prediction error. A change in therapy during the prediction period or a worsening of the patient’s condition may lead to incorrect predictions. Therefore, prediction should be considered as a regression model that simulates the current dynamics of patient characteristics and the impact of treatment in the estimation window for unchanged future fluid input.

Statistical analyses of the prediction windows of the entire patient cohort revealed a mean RMSE of slightly less than 1 L when a sufficient amount of time series data (48 h or more) is provided to the model. As can be seen in Figure 6B, the shape of the mean RMSE values shows a slightly U-shaped trajectory. This may indicate that an estimation time span of 24 h is not sufficient for correct patient characterization. The prediction accuracy becomes noticeably worse when using estimation windows of 120 h or longer. A possible reason is the use of the broken stick model with the assumption of a constant fluid intake after the estimated turning point although the fluid intake changes over time with changes in the patient’s current hydration status. In addition, the patient characteristics change dynamically over time and therefore the estimation window should be chosen to be long enough for parameter identification, but short enough to only include very recent data to reflect the current state of the patient as good as possible. With a prediction time of 8 h, almost 50% of CFB predictions are within the range of ±0.5L, and 77% are still within the clinically acceptable range of ±1.0L, demonstrating good clinical applicability by optimizing fluid status of the patient in a critical care situation.

The model presented only estimates the behavior of the entire patient system by analyzing fluid intakes and losses and does not simulate a mechanistic fluid compartment system. Distributions of the fluids in the compartments and changes over time are therefore not included. Increased fluid retention due to, for example, a sodium excess or the accumulation of fluids in edemas leads to changes that are usually not relevant in the prediction window and can be therefore partially neglected. However, it must be emphasized at this point that the goal of this work was to find an algorithm that could be used with the existing data in a realistic scenario in daily clinical practice and that does not require additional subjective assessments or other biomarkers.

In summary, the presented digital model is not based on machine or deep learning and therefore does not require large amounts of data for training and validation of the model. It allows for early recognition of a change in the CFB course, which leads to faster adjustment of treatment. The system theory model, based on single patient data, thus shows high potential for adapting and optimizing fluid management in critical care situations for a clinically feasible prediction window and could be further developed into a decision support system for fluid balance and imbalance profiling. With the here presented approach it is therefore possible to support fluid management with already existing data - with the mentioned limitations. Its advantage is its ability to reduce workload by, for example, taking over complex calculations or informing responsible clinicians of trends in changing health parameters.

Additional clinical studies are needed to further improve the presented approach in terms of methodological accuracy and clinical validity by considering additional relevant clinical factors. The next steps toward a decision support system to assist clinicians with fluid management are the introduction of a feedback mechanism. The use of a dynamic, non-linear function instead of the presented TF and its embedding in a semi-closed loop could allow both the estimation of CFB trend in response to different fluid intake regimes and support the identification of the most appropriate CFI trajectory to achieve targeted CFB goals when treating individual patients.

## Data Availability

The data analyzed in this study is subject to the following licenses/restrictions: The data set is not adequately anonymized and therefore allows for traceability of patient information. The dataset is not publically available.
